# Carrion’s Disease: More Than a Sand Fly–Vectored Illness

**DOI:** 10.1371/journal.ppat.1005863

**Published:** 2016-10-13

**Authors:** Maria J. Pons, Cláudia Gomes, Juana del Valle-Mendoza, Joaquim Ruiz

**Affiliations:** 1 Research Center and Innovation of the Health Sciences Faculty, Faculty of Health Sciences, Universidad Peruana de Ciencias Aplicadas (UPC), Lima, Peru; 2 ISGlobal, Barcelona Ctr. Int. Health Res. (CRESIB), Hospital Clínic, Universitat de Barcelona, Barcelona, Spain; 3 Instituto de Investigación Nutricional, Lima, Peru; Nanyang Technological University, SINGAPORE

## What Is Carrion’s Disease?: The Forgotten

Carrion’s disease is a biphasic illness (S1 Fig) caused by an infection of *Bartonella bacilliformis*, a bacterium that is transmitted through bites of certain phlebotomine sand flies in the Andean valleys of Peru and in some areas of Ecuador and southern Colombia [1,2]. The acute phase, called Oroya fever, is a serious, life-threating illness that mainly affects immunologically naïve populations, such as children. It is also of special concern in pregnant women, because high mortality rates have been described as well as miscarriages, preterm births, and fetal deaths [3]. In this acute phase, the absence or delay of antibiotic treatment may lead to fatal outcomes. In fact, it is considered that, in the pre-antibiotic era, the lethality of this illness ranked between 40% and 88% [1,2]. In the chronic phase, classically considered to occur in previously exposed inhabitants, *B*. *bacilliformis* induce endothelial cell proliferation, producing skin lesions called Peruvian warts. In this phase, the lethality is very low [1]. Additionally, the presence of asymptomatic carriers is frequent, although the real numbers remain uncertain because of the difficulty in detecting these subjects (Table 1) [4].

**Table 1:** Main facts on Carrion's disease.

Facts^1^	Oroya fever	Asymptomatic carrier	Peruvian wart
Symptoms and Signs	Abdominal pain		Arthralgia
	Anorexia/Hiporexia		Bone pain
	Arthralgia		Fever
	↑ Bilirubin		Headache
	Chills 1		Joint pain
	Diarrhea		Lymphadenopathy
	Dyspnea		Malaise
	**Fever (up to 99%)** ^**2**^		Myalgia
	Headache		**Skin lesions**
	**↓ Hematocrit (up to >80%)**		
	**Hemolytic Anemia (up to >90%)** ^**3**^		
	Hepatomegaly		
	Hypothermia		
	Jaundice		
	Lymphadenopathy		
	Malaise		
	Myalgia		
	Nausea/Vomiting		
	Pallor		
	**Pollakiuria**		
	↑ Protein C Reactive		
	Splenomegaly		
	Sweats		
	Systolic murmur		
	Tachycardia		
Complications	**Immunosuppression**		Bleeding
	***Co-infections*** ^***4***^		Dermal infection
	bloodstream *Salmonella*		Necrosis
	bloodstream *S*. *aureus*		
	Leptospirosis		
	***Latent infections*** ^***4***^		
	Histoplasmosis		
	Toxoplasmosis		
	Tuberculosis		
	Cardiovascular		
	*Anasarca*		
	*Cardiovascular shock*		
	*Congestive heart failure*		
	*Myocarditis*		
	*Pericardial effusion*		
	*Pericardial tamponade*		
	Gastrointestinal		
	*Digestive hemorrhage*		
	Gyneco-obstetrics		
	***Fetal death***		
	***Miscarriages***		
	***Pre-term births***		
	Hepatical		
	*Acute cholecystitis*		
	*Hepatocellular necrosis*.		
	Neurological		
	*Altered Mental Status*		
	*Increased intracranial pressure*		
	*Coma*		
	*Convulsion*		
	Respiratory		
	*Acute Pulmonary Edema*		
	Other		
	*Purpura*		
	*Renal insufficiency*		
Treatment	Blood Transfusions	Amoxicillin plus clavulanic acid^5^	Erythromycin, Azithromycin
	Amoxicillin plus clavulanic acid^5^	Ciprofloxacin	Rifampicin
	Chloramphenicol ± other antibiotics		
	Ciprofloxacin ± Cephalosporin		
Outcome	Development of partial immunity^6^	Perpetuators of the illness	Development of partial immunity
	Lack of bacterial clearance^6^	Potential infected blood/organ donations	Lack of bacterial clearance
	Vertical transmission risk	Vertical transmission risk	Vertical transmission risk
	Death^7^		

In bold, the more frequent and/or relevant signs and symptoms, as well as complications. Orange shaded, complications that more often result in a fatal outcome.

^1^ Nonexhaustive list.

^2^ Moderate (usually less than 39°C) and intermittent.

^3^ With negative Coombs test.

^4^ Listed some of the most commonly detected.

^5^ Mainly in pregnant women.

^6^ Uncertain number due to the low blood bacterial burden and the lack of sensitive diagnostic tools. It has been reported that 45% of inhabitants from an endemic area have antibodies against *B*. *bacilliformis* [2].

^7^ If correctly treated, case fatality rates ranks from 0.5%–1% in peripheral health centers and 8%–10% in reference centers (because of the reception of complicated cases); when not treated, case fatality ranks between 40% and 88%. In any case, it is especially relevant among children and pregnant women.

Until now, no reservoir other than humans has ever been described, and, thus, due to the geographically defined area and the lack of animal reservoirs, this disease could be potentially eradicated.

The role of undescribed *Bartonella* spp. as a cause of Carrion’s-disease–like symptoms cannot be ruled out. Indeed, other *Bartonella* spp. have correlated to Carrion’s-disease–like presentations. Thus, *Bartonella rochalimae*, which is disseminated worldwide [5], was associated with a mild Oroya-fever–like episode in a tourist after a trip to Peru, whereas *Bartonella ancashensis* has been isolated from Peruvian warts of children living in an endemic Peruvian area [1].

**Fig 1:**
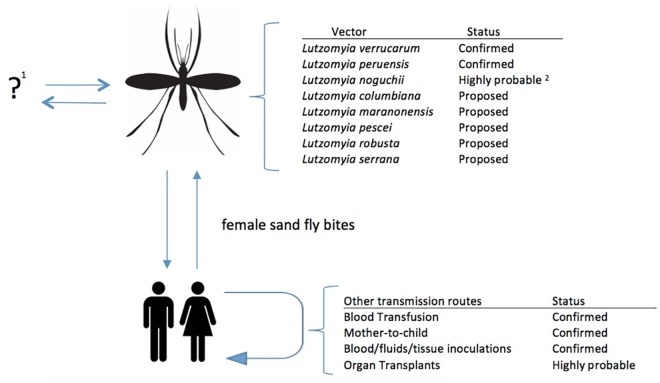
*B*. *bacilliformis* transmission routes. ^1^ Humans are the only known reservoir. ^2^ Despite a report by Noguchi et al [6], no confirmatory result has been published.

Despite vector transmission being by far the most relevant route of transmission, other possible routes should be highlighted. Due to the nature of the illness, all direct inoculation or contact with infected human blood may result in its acquisition; thus, blood transfusions as well as accidental contact with infected blood in laboratories or during medical practices need to be considered. Additionally, vertical transmission and contact with other human fluids should also be considered (Fig 1).

## Vector Transmission of *B*. *bacilliformis*: The Story Always Told

Native inhabitants traditionally considered sand flies as causing Carrion’s disease, and, in the middle of the 18th century, Cosme Bueno proposed their role as vectors of Carrion’s disease and *Leishmania* [7]. Nonetheless, the vector role of *Lutzomyia verrucarum* in the illness was not confirmed until 1913, more than 40 years after the devastating Carrion’s disease episode in 1870–1871 that occurred during the construction of the Lima-La Oroya railway, causing several thousands of deaths [1,8].

*B*. *bacilliformis* is naturally transmitted by sand flies belonging to the *Lutzomyia* genus, mainly *L*. *verrucarum*, but also *Lutzomyia peruensis* [1,2]. However, the illness is also present in areas where these vectors are absent, suggesting the presence of undescribed vectors, or the undetected established vectors of *B*. *bacilliformis* [1]. In fact, the vector role of *L*. *peruensis* was described after an outbreak in an *L*. *verrucarum*-free area [1].

Vector sand flies are present in the inter-Andean valleys between 400 and 3,200 m [1,2]. Nonetheless, since the late 1990s, a continuous expansion of the illness to previously considered free areas, including coastal and high jungle areas, has been evident. It has been associated with the current situation of climate change, which, together with human activities, is presumably affecting the vector distribution, leading to vector expansion [1]. Additionally, the El Niño phenomenon results in increasing humidity levels, favoring sand fly reproduction and facilitating the development of Carrion’s disease outbreaks [1]. Moreover, the potential of Lutzomyia spp. living in non-endemic areas to become adapted as vectors of *B. bacilliformis* should not be ruled out.

## Blood Transfusion: The Hidden Risk

Similar to other *Bartonella* species [9], *B*. *bacilliformis* is able to survive a long time in infected blood at 4°C [10,11]. In 1926, Noguchi reported that *B*. *bacilliformis* can survive 152 days in experimentally infected monkey blood samples stored at 4°C [10]. More recently, viable *B*. *bacilliformis* was recovered from Oroya fever patients’ blood stored at 4°C for as long as 30 months [11]. It has been considered that infections with *B*. *bacilliformis* occur in populations unlikely to be qualified as blood donors [12]. Nonetheless, the slow bacterial growth, the inability of definitive diagnostic approaches to consistently detect carriers [4], and the undefined duration of the asymptomatic carrier status, which may be up three years [13], are clear risks that may result in posttransfusion infections. Moreover, this risk, which is not limited to endemic regions, may extend to other areas due to the migration phenomena from rural to urban areas and low- or middle- to high-income countries.

In endemic zones, blood banks perform blood smears to detect the presence of *B*. *bacilliformis*, while in neighboring regions, blood donations of persons living in endemic areas are not accepted [14]. In blood banks from other regions or countries, no specific measures are taken to determine the presence of *B*. *bacilliformis*. Nonetheless, confirmed or suspected cases of post-transfusion Carrion’s disease are scarce. This may be due to the low-income nature of the main regions affected, which results in a low number of cases being reported, as well as a high number of previously exposed inhabitants, of up to 45% [2], who have developed partial immunity that may prevent or minimize the effect of transfusion-mediated transmission. In fact, earlier 20th century reports considered that almost all local inhabitants had partial immunity [8]. Moreover, most data on Carrion’s disease are only reported at a local level, thereby contributing to lack of disease visibility at an international level.

From the few reports present in the literature, two are strongly suggestive of transfusion transmission. In 1972, a newborn died from Oroya fever after a blood transfusion in a *B*. *bacilliformis*-endemic area. In this case, vertical transmission was not considered, as the mother did not have a previous *Bartonella* infection [15]. More recently, the acquisition of Oroya fever has been described in a chronic myeloid leukemia patient receiving multiple blood and platelet transfusions, one of which was contaminated with *B*. *bacilliformis* [14].

The possibility of *B*. *bacilliformis* transmission during organ transplantation is also plausible. Indeed, posttransplant infection with a Bartonellaceae (*Bartonella henselae*) has been proposed [16].

## Vertical Transmission: The Nightmare

Vertical transmission was first proposed in 1858 by Tomas de Salazar [15]. Similarly, in 1913, Strong et al stated “We saw cases in young nursing children, and Campodonico and Monge state it occurs in newly born infants,” again suggesting the presence of vertical transmission [8]. Subsequently, both Malpartida and Colareta proposed the same in the mid-1930s [15].

Nonetheless, unequivocal reports in the recent literature are scarce. In addition to the reasons for the low number of reports of transfusion transmission, the serious consequences of the acute phase of Carrion’s disease during pregnancy should also be considered, especially when the infection occurs in the first months of pregnancy, affecting both the mother with serious complications and high lethality for the fetus, including preterm births, miscarriages, or fetal deaths, among others [3,15]. Additionally, social attitudes and traditional practices may lead to delays or nonuse of health centers during pregnancy or after child birth [17], resulting in underestimation or misdiagnosis of mother-to-child *B*. *bacilliformis* transmissions, which thus may be higher than considered.

In 2003, it was reported that blood samples collected from a preterm child, 90 minutes after birth, of a mother with verrucous lesions, resulted in *B*. *bacilliformis*-positive culture [15]. In 1994, a neonate (19 days) was reported with Oroya fever, in whom 30% of the red blood cells were infected, thereby implicating the mother with general malaise, and diagnosed with a positive blood smear [18]. Finally, in 2015, another case was reported in which a 22-day-old child of a mother with a Peruvian wart was admitted with Oroya fever [19].

## Intentional or Accidental Inoculation with Fluids or Blood: Heroes, Crazies, and Unfortunates

Inoculation with contaminated body fluids is a direct way to acquire Carrion’s disease. Although currently direct human inoculations with infected human fluids are limited to accidents, in the last years of the 19th and in the early 20th centuries, experiments inoculating volunteers with either infected blood or wart exudates were performed. The most classical example is the self-inoculation of Daniel Alcides Carrión in 1885. He inoculated himself with a wart exudate and developed Oroya fever with a fatal outcome [20]. Subsequently, in 1928, Garcia Rosell received an accidental inoculation from the contaminated blood of a patient with Oroya fever and developed a febrile illness that was cured, followed by eruption of Peruvian warts [20].

## Conclusion: Risks and Opportunities

Although Carrion’s disease is currently restricted to specific geographical zones, increasing tourism to endemic regions together with continuous human migratory processes may lead to both imported cases and presence of asymptomatic carriers outside of traditional areas, driving towards establishment of non-vectorial *B*. *bacilliformis* mother-to-child transmission or through blood transfusions. Those, along with the incessant and growing movement of goods, may also facilitate the accidental introduction of vectors into atypical habitats. These findings highlight the risk of *B*. *bacilliformis* transmission beyond traditionally affected regions and reinforce the need to develop a Carrion’s disease eradication agenda.

## Supporting Information

S1 Fig *B*. *bacilliformis* may infect three different populations: healthy never exposed, healthy pre-exposed, or asymptomatic carriers (the infection of persons with either active Oroya fever or Peruvian wart is not considered, because these people should be under treatment). The illness evolution may vary leading to Oroya fever, Peruvian wart, or asymptomatic infection with different easiness. Moreover, although no data are available, the natural bacteria clearance may not be ruled out. Although not to scale, the arrows’ size represents the probability of infection evolution.? ^1^ No data about.
(DOCX)
