# Supplementary material for: Carrion’s Disease: More Than a Sand Fly–Vectored Illness
Source: PLoS Pathog. 2016 Oct 13;12(10):e1005863. doi: 10.1371/journal.ppat.1005863 (PMC5063350; doi:10.1371/journal.ppat.1005863)
Supplement: S1 Fig — The illness evolution may vary leading to Oroya fever, Peruvian wart, or asymptomatic infection with different easiness. Moreover, although no data are available, the natural bacteria clearance may not be ruled out. Although not to scale, the arrows’ size represents the probability of infection evolution.? 1 No data about. (DOCX) [file ppat.1005863.s001.docx]

Infections routes

Infection by *B. bacilliformis*

- Infected *Lutzomyia* bite
- Vertical Transmission
- Blood Transfusion
- Infected blood/fluid contact
- Others?

Asymptomatic carrier

Healthy pre-exposed

LBC

Infected Population

? ^1^

Healthy never exposed

Infection evolution

Illness phases

Peruvian Wart

Asymptomatic carrier

Oroya Fever
